# Thermal gradient ring reveals thermosensory changes in diabetic peripheral neuropathy in mice

**DOI:** 10.1038/s41598-022-14186-x

**Published:** 2022-06-13

**Authors:** Sachiko Sasajima, Masaki Kondo, Nobuhiko Ohno, Tomoyo Ujisawa, Mikio Motegi, Tomohide Hayami, Saeko Asano, Emi Asano-Hayami, Hiromi Nakai-Shimoda, Rieko Inoue, Yuichiro Yamada, Emiri Miura-Yura, Yoshiaki Morishita, Tatsuhito Himeno, Shin Tsunekawa, Yoshiro Kato, Jiro Nakamura, Hideki Kamiya, Makoto Tominaga

**Affiliations:** 1 Division of Diabetes, Department of Internal Medicine, Aichi Medical University School of Medicine, 1-1 Yazakokarimata, Nagakute, Aichi 480-1195 Japan; 2 Division of Cell Signaling, National Institute for Physiological Sciences, 5-1 Higashiyama, Myodaiji-cho, Okazaki, Aichi 444-8787 Japan; 3 Department of Anatomy, Division of Histology and Cell Biology, School of Medicine, Jichi Medical University, 3311-1 Yakushiji, Shimotsuke, Tochigi 329-0498 Japan; 4 Division of Ultrastructural Research, National Institute for Physiological Sciences, 5-1 Higashiyama, Myodaiji-cho, Okazaki, Aichi 444-8787 Japan; 5 Thermal Biology Group, Exploratory Research Center on Life and Living Systems (ExCELLS), 5-1 Higashiyama, Myodaiji-cho, Okazaki, Aichi 444-8787 Japan; 6 Department of Innovative Diabetes Therapy, Aichi Medical University School of Medicine, Nagakute, Japan; 7 Department of Physiological Sciences, Sokendai, Okazaki, Japan

**Keywords:** Neuroscience, Physiology

## Abstract

Diabetic peripheral neuropathy (DPN) includes symptoms of thermosensory impairment, which are reported to involve changes in the expression or function, or both, of nociceptive TRPV1 and TRPA1 channels in rodents. In the present study, we did not find changes in the expression or function of TRPV1 or TRPA1 in DPN mice caused by STZ, although thermal hypoalgesia was observed in a murine model of DPN or TRPV1^**−/−**^ mice with a Plantar test, which specifically detects temperature avoidance. With a Thermal Gradient Ring in which mice can move freely in a temperature gradient, temperature preference can be analyzed, and we clearly discriminated the temperature-dependent phenotype between DPN and TRPV1^**−/−**^ mice. Accordingly, we propose approaches with multiple behavioral methods to analyze the progression of DPN by response to thermal stimuli. Attention to both thermal avoidance and preference may provide insight into the symptoms of DPN.

## Introduction

Diabetic peripheral neuropathy (DPN) is a frequently observed complication of DM with one of the earliest onsets, and causes substantial morbidity. DPN is a chronic neurodegenerative condition, and is thought to progress in the order of sensory axons, autonomic axons, and then motor axons. Main symptoms of DPN include spontaneous pain (burning, lancinating, tingling, and shooting), hyperalgesia, allodynia, and loss of protective sensation^1^. However, the underlying mechanisms of DPN are complicated and various factors including high glucose levels, insulin levels, hyperlipidemia, and microangiopathy interact mutually to cause the metabolic abnormalities^2^.

Streptozotocin (STZ) is commonly used to create a model of type 1 DM in rodents^3,4^. Some studies using a STZ-induced model of diabetes have shown early thermal hyperalgesia and subsequent hypoalgesia^5–8^. Other studies have shown hypoalgesia without the early thermal hyperalgesia^9^. The symptoms observed in the model are similar to those seen in cases of DPN in humans^10^. Thermal hyperalgesia exhibits increased sensitivity to external stimuli, which may be caused by damage to nociceptors or peripheral nerves. By contrast, hypoalgesia is caused by degeneration and demyelination of myelinated sensory fibers, and dysfunction of nociceptive unmyelinated C fibers^2^. Although hypoalgesia is eventually caused by disruption of neural conduction circuits^11^, it is sometimes observed before histological degeneration or demyelination of nerve fibers. Interestingly, mechanisms of thermal hyperalgesia are reported to be correlated to chronic hypoalgesia^12^. These observations suggest that some of the factors involved in the mechanisms of hypoalgesia are the same as those found for hyperalgesia. Among the factors, changes in the expression and function of transient receptor potential (TRP) channels have been investigated.

Various types of TRP channels are expressed in sensory neurons. In particular, TRP vanilloid 1 (TRPV1)^13–15^, TRPV2^16,17^, TRPV4^18^, TRP ankyrin 1 (TRPA1)^19,20^, TRP melastatin 3 (TRPM3)^21,22^ are considered to be involved in nociception in rodents. Among them, TRPV1 and TRPA1 contribute strongly to nociception by unmyelinated C fibers. TRPV1 is activated by many kinds of nociceptive stimuli including capsaicin and protons^13–15^. TRPA1 is also activated by nociceptive stimuli including allyl isothiocyanate, cinnamaldehyde, formaldehyde, and ROS^19,20^. Interestingly, TRPV1^13–15^ and TRPA1^23,24^ are reported to be activated by noxious heat and cold stimulation, respectively, although the temperature sensitivity of mammalian TRPA1 remains controversial^25^. Temperature sensitivity of TRPV1 and TRPA1 might be associated with the symptoms of thermosensory impairment in DPN. Indeed, a transient increase in TRPV1-mediated responses in the primary sensory neurons has been observed during periods of thermal hyperalgesia^8,26,27^, but the responses declined during periods of thermal hypoalgesia^8,27^. At least one study found that expression of TRPV1 proteins in a hyperalgesic period increased, although decreased expression during hypoalgesia and changes in temperature-dependent phenotypes were not examined in the study^28^. TRPA1 channels are activated by methylglyoxal^29^ and ROS^30^, which are increased in a model of DPN in rodents. Impaired blood flow in a model of DPN in mice caused cold hyperalgesia in the initial painful stage, and DRG neurons in the mice showed increased TRPA1 activities^31^. STZ has neurotoxic effects which include increased levels of TRPV1 expression in the dorsal horn of the spinal cord^32^ and direct activation of TRPA1 by STZ^33^, with both effects occurring regardless of blood glucose levels. However, STZ is rapidly metabolized and cleared from the body within hours of administration^34^; accordingly, changes in expression and/or function of TRPV1 and TRPA1 upon STZ administration are unlikely to affect the chronic symptoms observed from several weeks to months after administration^35^.

It is impossible to determine the pain sensation in animals directly, and we can only predict their pain sensation through the responses to stimuli given to them, which are generally called “pain-related behaviors”. There are several types of behavioral tests for measuring pain-related behaviors; a pain susceptibility test to observe avoidance behaviors in response to acute stimuli and an association test including preference behaviors with stimuli and environmental circumstances^36^ etc. To evaluate the temperature-dependent phenotypes of DPN in mice, avoidance tests, such as a Plantar test^37,38^ and a hot-plate test^39^, have been widely used. These two assays are recognized to evaluate not only reflex behaviors by mice, but also avoidance behaviors upon detection of thermal nociceptive stimuli applied to their peripheral sensory neurons. However, outcomes might be affected in mice that are confined to a limited area. In addition, the temperature preference of mice might not be evaluated by the two types of avoidance assays, and temperature preference may be a component of temperature-dependent phenotypes observed in DPN. On the other hand, the association tests measure pain-related behavioral responses through pain avoidance and preference. The association tests allow us to evaluate more active mouse actions upon stimulus detection, and mice can learn and actively avoid the stimuli. With these tests we can evaluate pain-related responses not only as a result of avoidance, but also by preference of rodents, because mouse behavior can be determined by the balance between avoidance and preference, where the two factors interact mutually and are difficult to discriminate.

In the present study, we could not find the changes in the expression or function of TRPV1 or TRPA1 in a STZ-induced model of DPN in mice, although thermal hypoalgesia was similarly observed in the mouse model of DPN and TRPV1^−/−^ mice. We found a behavioral assay with Thermal Gradient Ring clearly discriminated temperature-dependent phenotypes between the DPN and TRPV1^**−/−**^ mice. Accordingly, we propose this new behavioral assay system that allows us to analyze the DPN phenotypes more in detail, and the system can be used for many types of temperature-dependent phenotypes.

## Results

### Changes in blood glucose levels and body weights in mice administered with STZ

**Figure 1:**
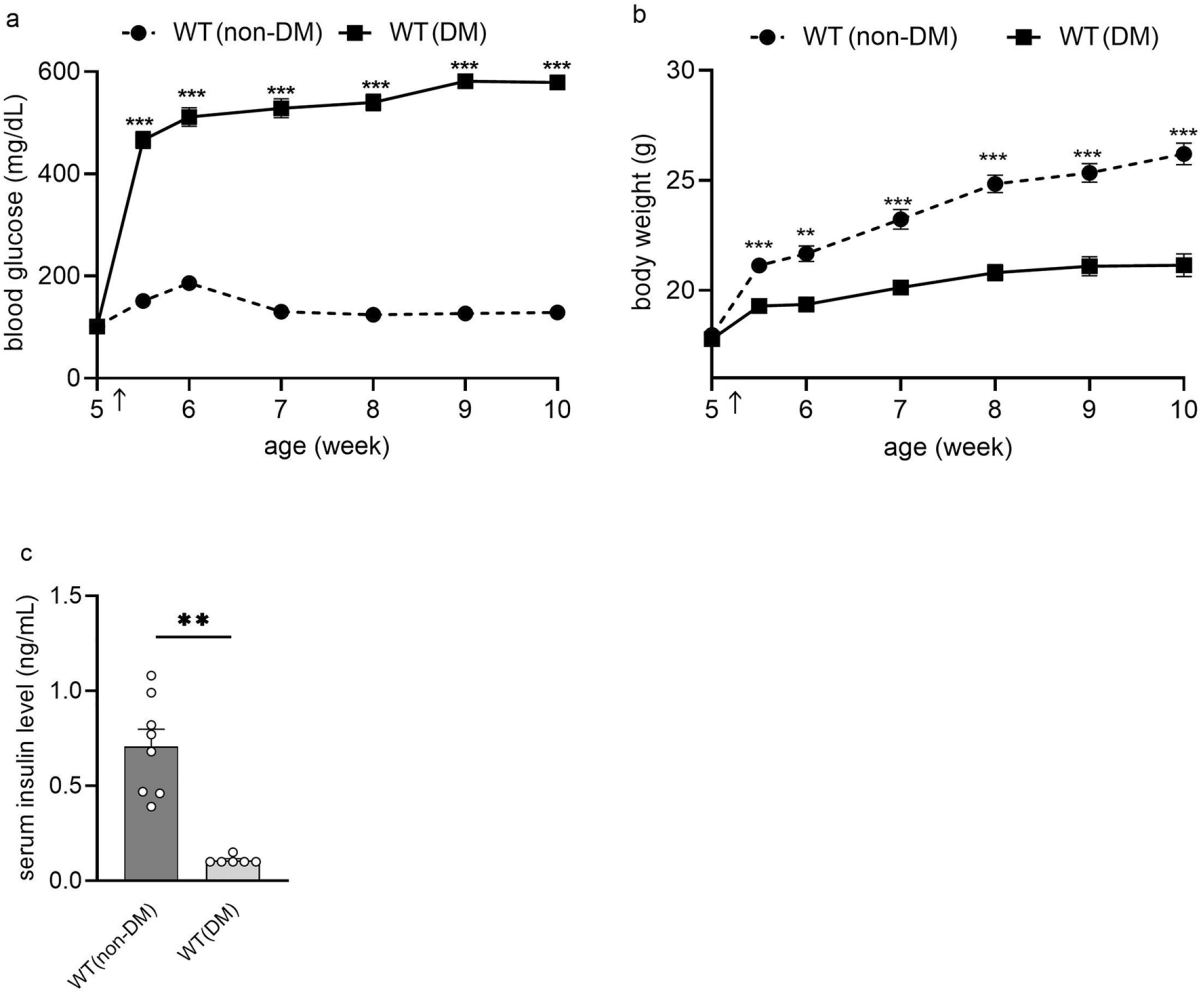
Changes in blood glucose levels, body weight, and serum insulin levels after administering STZ. (**a**) Changes in blood glucose levels before and after intraperitoneal injection of streptozotocin (STZ 150 mg/kg) or 0.02 M citrate buffer (pH 4.5) in 5-week-old wild-type mice (diabetes mellitus (WT (non-DM)), n = 10; non diabetic (WT (non-DM)), n = 10). (**b**) Changes in body weight of the same mice are shown in (**a**,**c**)**.** Comparison of serum insulin levels of WT (DM) and WT (non-DM) mice at 10 weeks old (WT (DM), n = 6; WT (non-DM), n = 8). Open circles indicate each insulin level. Timing for STZ and the buffer administration is indicated by arrows. The data are presented as the means ± SEM. Two-way repeated measures ANOVA followed by a Bonferroni post hoc test (**a**,**b**) and a two-tailed *t* test (**c**) was used for comparison. **p < 0.01, ***p < 0.001 vs. WT (non-DM).

STZ induces death of pancreatic beta cells after single administrations of 100–200 mg/kg in mice. Therefore, STZ is commonly used to induce type 1 diabetes in rodents to study DPN^3,4^. In the present study, 5–10-week-old male mice were used and observed up to 5 weeks after the onset of diabetes. When they were 5 weeks old, the mice were injected intraperitoneally with a single dose of STZ (150 mg/kg) after a 24-h fasting period. Blood glucose levels became more than 400 mg/dL within one week after the treatment. Mice that maintained high blood glucose levels throughout the course of the study compared with a wild-type nondiabetic (WT (non-DM)) mouse group were defined and used as the WT diabetic (WT (DM)) mouse group (Fig. 1a). WT (DM) mice gained less weight than WT (non-DM) mice (Fig. 1b). Serum insulin levels in mice 5 weeks after STZ administration almost decreased below the level of detection allowed by the sensitivity of the insulin assay, confirming that hyperglycemia was associated with a secretory deficiency of insulin (Fig. 1c).

TRPV1-deficient (TRPV1^**−/−**^) and TRPA1-deficient (TRPA1^**−/−**^) mice were used to determine whether either TRPV1 or TRPA1, or both, play a role in the phenotypes observed in DM mice. The body weights of TRPV1^**−/−**^ (non-DM) mice tended to be lower than those of WT (non-DM) mice (Supplementary Fig. 1a), in part because they were not littermates and age-matched WT (non-DM) mice were obtained from a vendor. However, blood glucose levels did not differ over time between the genotypes (Supplementary Fig. 1b). Furthermore, we administered STZ to TRPV1^**−/−**^ and TRPA1^**−/−**^ mice in the same methods as WT (DM) mice, and defined the hyperglycemic group as TRPV1^**−/−**^ (DM) and TRPA1 ^**−/−**^(DM) mice, respectively. TRPV1^**−/−**^ (DM) and TRPA1^**−/−**^ (DM) mice also showed an increase in blood glucose with STZ administration similar to WT (DM) mice (Supplementary Fig. 1c, d).

### Change in thermal pain sensitivity of WT (DM), TRPV1^−/−^ (non-DM) and TRPA1^−/−^(non-DM) mice

**Figure 2:**
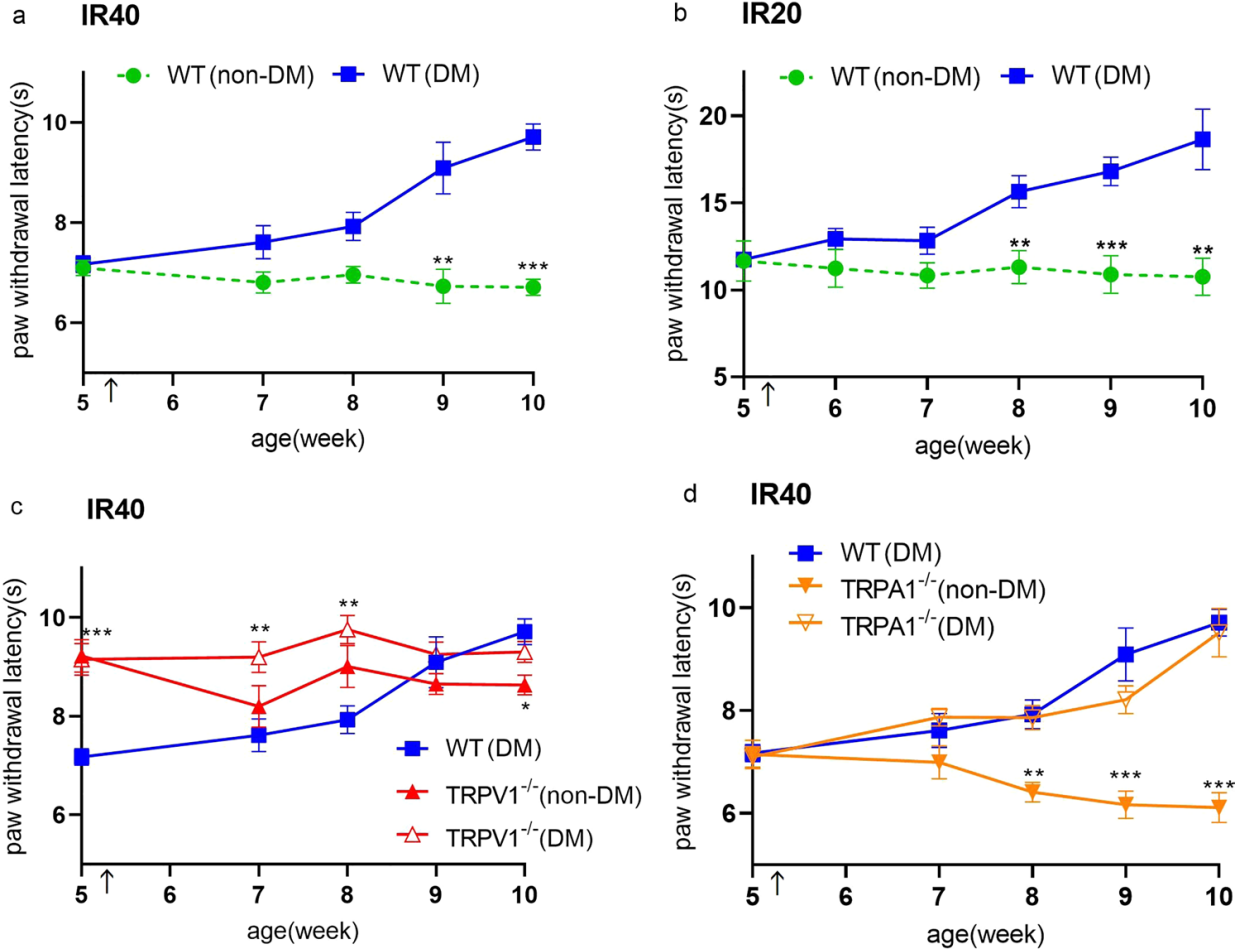
Changes in paw withdrawal latency in WT (DM), TRPV1^**−/−**^ (non-DM) and TRPA1^**−/−**^ (non-DM) mice. (**a,b**) Changes in paw withdrawal latency (PWL) by IR intensity 40 (**a**) and IR intensity 20 (**b**) before and after intraperitoneal injection of streptozotocin (STZ 150 mg/kg) or an equal volume of citrate-buffer vehicle in 5-week-old wild-type mice (diabetic mellitus (WT (DM)), n = 8–9; non-diabetic (WT (non-DM)), n = 10). (**c**) Comparison of PWL by IR intensity 40 in WT (DM) (n = 9), TRPV1^**−/−**^ (non-DM) (n = 10), and TRPV1^**−/−**^ ( DM) (n = 8) mice. (**d**) Comparison of PWL by IR intensity 40 in WT (DM) (n = 9), TRPA1^**−/−**^ (non-DM) (n = 9), TRPA1^**−/−**^ (DM) (n = 8) mice. The PWL at 5 weeks of age was measured a few days before STZ and the buffer administration. Timing for STZ and the buffer administration is indicated by arrows. The data are presented as box-and-whisker plots. Two-way repeated measures ANOVA followed by a Bonferroni post hoc test. *p < 0.05, **p < 0.01, ***p < 0.001 vs. DM.

Mice were tested for thermal pain sensitivity by measuring paw withdrawal latency (PWL) using a Plantar test. The PWL at 5 weeks of age was measured a few days before STZ or the buffer administration. First, we adjusted the infrared (IR) intensity to a PWL baseline of about 7 s (IR = 40, Fig. 2a) and conducted an experiment. We, then reduced the IR intensity (IR = 20, Fig. 2b) to detect subtle changes in thermal hyperalgesia, but could not define a hyperalgesia period. Therefore, we used the IR40 intensity in the following experiments. After STZ administration, the withdrawal latency of WT (DM) mice gradually increased for both IR intensities, and the difference compared with WT (non-DM) mice became statistically significant 3 to 4 weeks after STZ administration (corresponding to an age of 8 to 9 weeks) (Fig. 2a,b). We chose to use the WT (DM) mice 5 weeks after STZ administration because by then the mice showed thermal hypoalgesia. TRPV1^**−/−**^ (non-DM) mice were found to be insensitive to thermal stimuli in a Plantar test with significantly longer PWL through the trial compared with WT (non-DM) mice. PWL in TRPV1^**−/−**^ mice with and without STZ treatment was similar, and even before the STZ treatment PWL was significantly longer for TRPV1^**−/−**^ (non-DM) mice compared to WT (non-DM) mice (Fig. 2c). The PWL for TRPA1^**−/−**^ (non-DM) mice were similar to that for WT (non-DM) mice. TRPA1^**−/−**^ mice with STZ treatment (TRPA1^**−/−**^ (DM)) exhibited a similar time course to that observed in the WT (DM) mice (Fig. 2d).

### Changes in level of expression of TRPV1^−/−^ (non-DM) and TRPA1^−/−^ (non-DM) in DRG of WT (DM) mice

**Figure 3:**
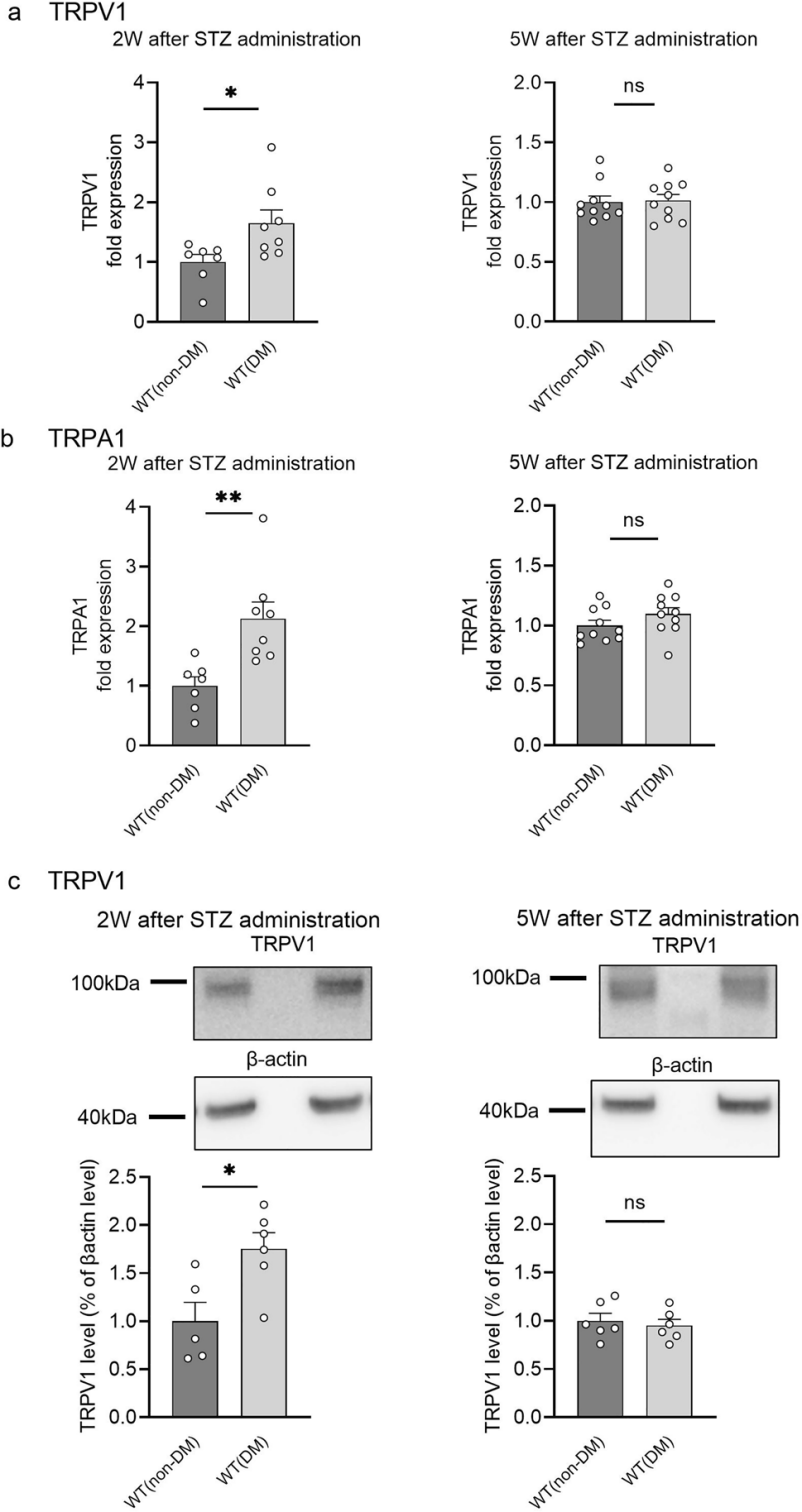
Expression of TRPV1 and TRPA1 in the dorsal root ganglia of mice 2 and 5 weeks after STZ administration. (**a,b**) mRNA expression levels of TRPV1 (**a**) and TRPA1 (**b**) in dorsal root ganglia (DRG) at 2 (left) and 5 (right) weeks after STZ administration. We used 36B4 as a housekeeping gene to normalize the expression. WT (non-DM), n = 7–10. WT (DM), n = 8–10. (**c**) Comparison of protein levels of TRPV1 in DRG by western blot analysis at 2 (left) and 5 (right) weeks after STZ administration. We used β-actin as an internal loading control. The predicted molecular weights of TRPV1 and β-actin are 95 and 42 kDa, respectively. WT (non-DM), n = 5–6. WT (DM), n = 6. The data are presented as the means ± SEM. A two-tailed *t* test was used for comparisons. *p < 0.05 and **p < 0.01. ns = not significant. Open circles indicate data.

After behavioral tests, we evaluated the expression of TRPV1 and TRPA1 in DRG of WT (DM) mice at two time points to determine receptor association with DPN. We chose 2 and 5 weeks after STZ administration, which correspond to before and after exhibiting thermal hypoalgesia, respectively (Fig. 2). Expression of both TRPV1 and TRPA1 mRNA was significantly greater in WT (DM) mice than in WT (non-DM) mice 2 weeks after STZ administration, while the difference was not significant 5 weeks after STZ administration (Fig. 3a,b).Protein levels of TRPV1 were significantly higher in WT (DM) mice at 2 weeks after STZ administration, but the difference was not significant at 5 weeks after STZ administration (Fig. 3c, Supplementary Fig. 2), showing changes at the level of protein similar to those of mRNA expression. We could not perform a western blot experiment for TRPA1 because to our knowledge there is no specific antibody available that would allow us to determine the protein levels of TRPA1 accurately.

### Functional evaluation of DRG neurons by Ca^2+^ imaging

**Figure 4:**
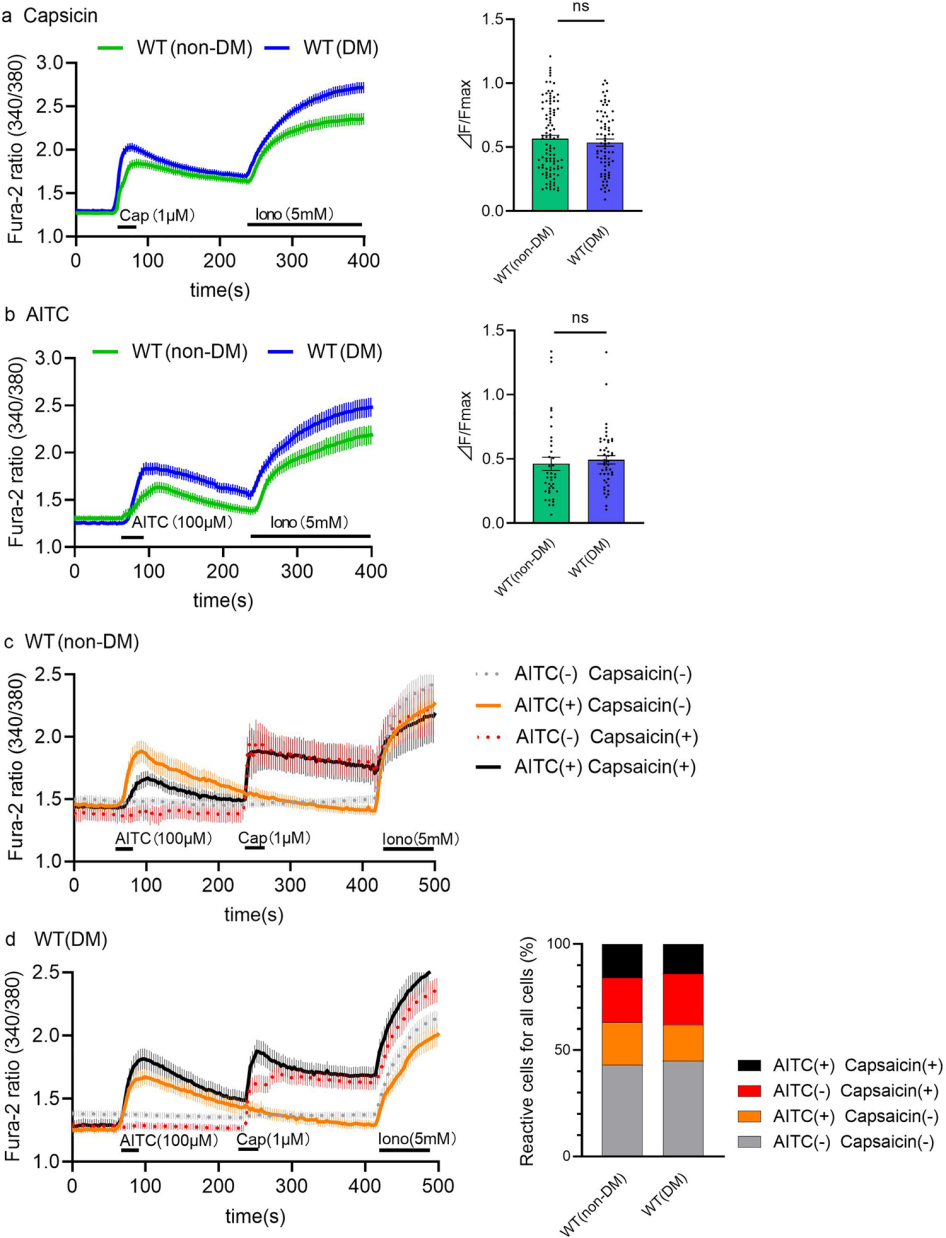
Functions of TRPV1 and TRPA1 in the dorsal root ganglia of mice 5 weeks after STZ administration. (**a,b**) (Left) Traces of mean Fura-2 ratios corresponding to intracellular Ca^2+^ concentrations in DRG neurons upon application of capsaicin (Cap) (1 μM, (**a**) in WT (non-DM) (green, n = 100 cells from 6 mice) or WT (DM) (blue, n = 74 cells from 5 mice), or allyl isothiocyanate (AITC) (100 μM, (**b**) in WT (non-DM) (green, n = 39 cells from 5mice) or WT (DM) (blue, n = 47 cells from 5mice) mice. (Right) Comparison of the values of the capsaicin-evoked Ca^2+^ responses (ΔF)/ionomycin-evoked Ca^2+^ responses (F_max_) (normalized ratios). (**c,d**) Traces of mean Fura-2 ratios in DRG neurons from WT (non-DM) (**c**, n = 104 cells from 5mice) and WT (DM) (**d**, n = 177 cells from 5mice) mice in response to application of capsaicin (1 μM) or AITC (100 μM). There are 4 kinds of neurons: no responses to both chemicals (AITC (−) Capsaicin (−), gray dotted line), responses to AITC alone (AITC (+) Capsaicin (-), orange solid line), responses to capsaicin alone (AITC (−) Capsaicin (+), red dashed line), and responses to both AITC and capsaicin (AITC (+) Capsaicin (+), black solid line). (**e**) Distribution of the 4 kinds of cells is shown in (**c** and **d**). The data are presented as the means ± SEM. A two-tailed *t* test was used for comparison. ns = not significant. Dots indicate each cell.

Because we found mRNA levels of TRPV1 and TRPA1 and protein levels of TRPV1 were not significantly different between WT (non-DM) and WT (DM) mice at 5 weeks after STZ administration, TRPV1and TRPA1 channel function was evaluated using a Ca^2+^-imaging method with Fura-2. Both capsaicin and allyl isothiocyanate (AITC) evoked a similar increase in intracellular Ca^2+^ concentrations in DRG neurons isolated from WT (DM) and WT (non-DM) mice (Fig. 4a,b). Although the experiments were performed using a same method for both groups, Fura-2 ratios looked higher in WT (DM) DRG neurons than WT (non-DM) neurons. The difference could be due to the changes in cell conditions during the experiment (Fig. 4a,b left). Therefore, we normalized the responses to the responses to ionomycin, and found no significant difference between the two groups (Fig. 4a,b right). To examine the possibility that the distribution of TRPV1 or TRPA1-expressing neurons, or both, are affected in WT (DM) mice, we compared the population of DRG neurons in response to AITC and capsaicin between the WT (non-DM) and WT (DM) mice. We classified DRG neurons into 4 groups: neurons that do not respond to either molecule (AITC (−) Capsaicin (−)), those that respond to AITC alone (AITC (+) Capsaicin (-)), capsaicin alone (AITC (−) Capsaicin (+)), or both (AITC (+) Capsaicin (+)) (Fig. 4c,d). There was no difference in the population size of TRPV1 alone, TRPA1 alone-expressing neurons, or both, between the WT (non-DM) and WT (DM) mice 5 weeks after STZ administration (Fig. 4e). The Fura2 ratios for neurons that were sensitive to AITC (+) Capsaicin (+) apparently differed between WT (non-DM) and WT (DM) mice. However, the difference was not statistically significant when normalized to the ratios caused by ionomycin (Supplementary Fig. 3). These data suggested that function and distribution of both TRPV1 and TRPA1 are unaffected by STZ-induced DM, at least at 5 weeks after administration.

### Analysis of nerve structure using an electron microscope

To investigate whether the WT (DM) mice had neurostructural changes 5 weeks after STZ administration, the sciatic nerves of WT (non-DM), WT (DM), and TRPV1^**−/−**^ (non-DM) mice were removed, and structural analyses were performed using light (LM) and transmission electron (TEM) microscopy. We observed no apparent difference in myelin structure among WT (non-DM), WT (DM) and TRPV1^**−/−**^ (non-DM) mice by either light or electron microscopy (Supplementary Fig. 4a). In addition, the axon diameters and g-ratios (the ratio of the axonal diameter to the diameter of the total axon including the myelin) were similar (Supplementary Fig. 4b–d). These results suggest that axonal and myelin structures were maintained in the WT (DM) mice 5 weeks after STZ administration and in TRPV1^**−/−**^ (non-DM) mice.

### Thermal Gradient Ring experiment

**Figure 5:**
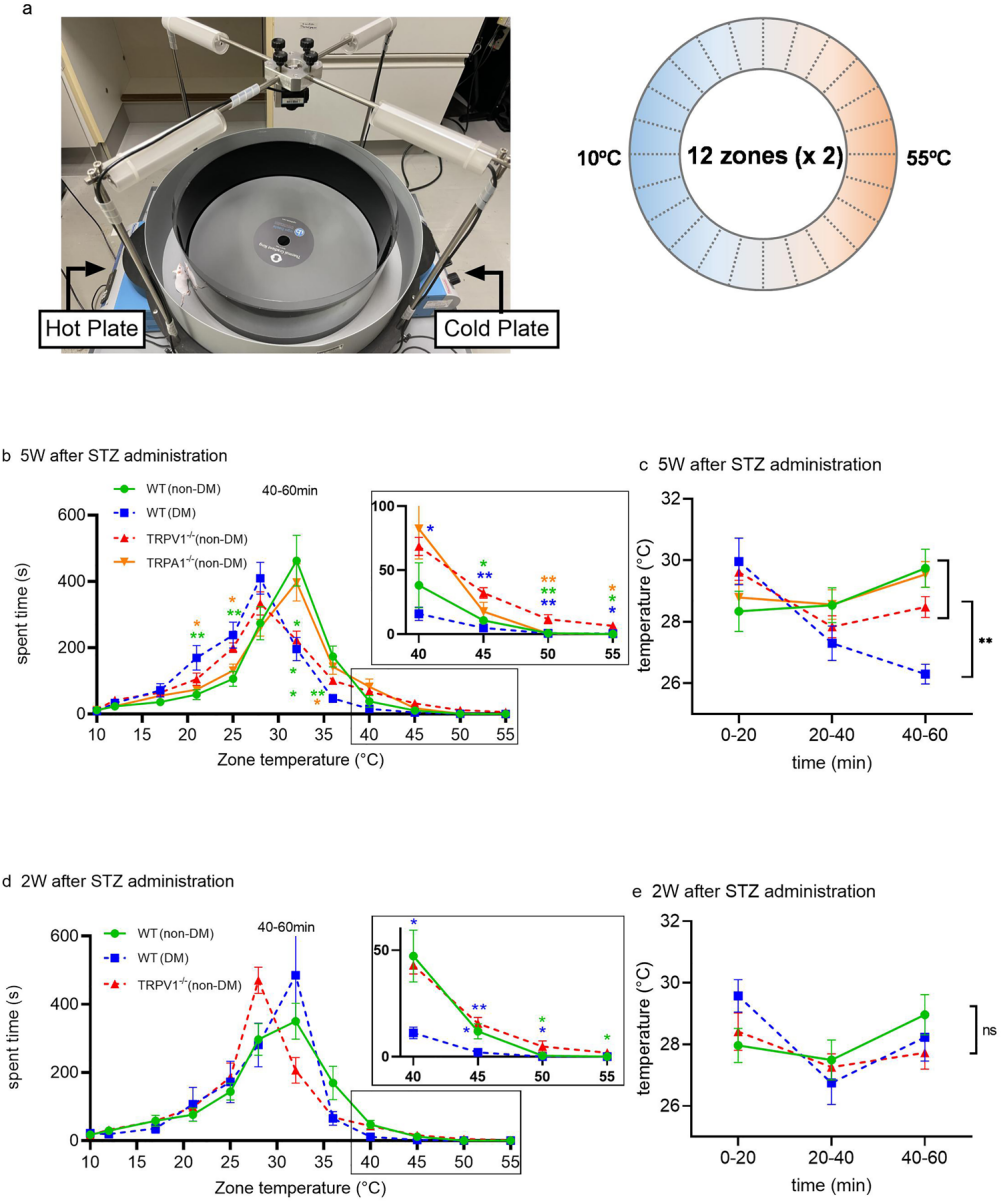
Thermal behavior assay with the Thermal Gradient Ring. (**a**) Image showing the Thermal Gradient Ring with a mouse (left) and an image of the floor of the Thermal Gradient Ring with 24 zones from 10 to 55 °C (right). (**b**) Comparison of “spent time” of 4 groups (WT (DM) mice 5 weeks after STZ administration (n = 10), and WT (non-DM) (n = 10), TRPV1^**−/−**^ (non-DM) (n = 10), TRPA1^**−/−**^ (non-DM) (n = 9) mice at the same age) during 40–60 min in the 60 min trial. Expanded data at 40–55 °C zones are also shown (**b**, right). (**c**) Mean values of preferred temperatures calculated from “spent time” and zone temperatures of (**b**), in the first, mid, and last 20 min. (**d**) Comparison of “spent time” of 3 groups (WT (DM) mice 2 weeks after STZ administration (n = 8), and WT (non-DM) (n = 10), TRPV1^**−/−**^ (non-DM) (n = 5) mice at the same age) during 40–60 min in the 60 min trial. Expanded data at 40–55 °C zones are also shown (**b**, right). (**e**) Mean values of preferred temperatures calculated from “spent time” and zone temperatures of (**d**), in the first, mid and last 20 min. The data are presented as the means ± SEM. A one-way ANOVA followed by a Bonferroni post hoc test was used for comparisons. *p < 0.05 and **p < 0.01. ns = not significant.

As indicated above, we could not observe any significant difference in TRPV1 amount or function, or distribution of TRPV1 and TRPA1 between WT (DM) and WT (non-DM) mice 5 weeks after STZ administration, which apparently contradicts the difference in thermal hypoalgesia observed between WT (DM) and TRPV1^**−/−**^ (non-DM) mice using a Plantar test. Therefore, we chose to use a Thermal Gradient Ring method to analyze the temperature-dependent behavior of the mice^40,41^. The floor of the ring is divided into 24 zones from cold to hot with the same temperature in two adjacent zones so that mice can move freely depending of their temperature preference or avoidance. We set a temperature gradient from 10 to 55 °C on the floor (Fig. 5a). We placed mice in the ring for 60 min and observed their movement continuously. We compared the “spent time” in each zone. For the analysis, the observation time of the 60 min trial was divided into three equal parts, and each 20 min segment was analyzed. First, we used WT (DM) mice (5 weeks after STZ administration that showed significant hypoalgesia in the Plantar test), WT (non-DM) mice, TRPV1^**−/−**^ (non-DM) mice and TRPA1^**−/−**^ (non-DM) mice at the same age. In the first 20 min (dotted lines), all of the mice (WT (non-DM), WT (DM), TRPV1^**−/−**^ (non-DM), and TRPA1^**−/−**^ (non-DM)) showed active exploratory behaviors, which reduced the time spent in any one zone. However, over time, mice gradually tended to stay in fixed zones (Supplementary Fig. 5). Therefore, we chose to analyze the data at 40–60 min to determine the “preference temperature” accurately. When spent time of 40–60 min was compared for all groups (Fig. 5b), temperature zones in which TRPA1^**−/−**^ (non-DM) mice preferred temperature zones similar to those for WT (non-DM) mice. Both WT (DM) and TRPV1^**−/−**^ (non-DM) mice appeared to prefer zones with lower temperatures than those preferred by WT (non-DM) mice. However, they clearly behaved differently in the high temperature zones of 40–55 °C, into which WT (DM) mice did not enter, while TRPV1^**−/−**^ (non-DM) did (enlarged inset in Fig. 5b). The TRPV1 channel is activated by noxious high temperatures, and it is easy to envisage that TRPV1^**−/−**^ (non-DM) mice spend longer times in the high-temperature zones. Although WT (DM) mice are believed to be insensitive to high temperatures as shown in the Plantar test, like TRPV1^**−/−**^ (non-DM) mice, spent time of WT (DM) mice in the high temperature zones was not significantly different from that of WT (non-DM) mice. Next, preference temperature for each temperature zone at 40–60 min was calculated as the mean value of the spent time in each temperature zone (Fig. 5c). WT (DM) mice showed a preference temperature of around 26 °C which was significantly lower than the temperature preferred by the other mice (about 28–30 °C). When the behaviors at 40–55 °C were analyzed, we found WT (DM) mice clearly avoided zones with temperatures over 45 °C.

We performed experiments with mice 5 weeks after STZ stimulation because mice at that age showed clear thermal hypoalgesia with a Plantar test (Fig. 2a). Then, a similar study was performed using mice 2 weeks after STZ administration (7 weeks old), which showed no significant behavioral difference in the Plantar test, and WT (non-DM) and TRPV1^**−/−**^ (non-DM) mice of the same age (Fig. 5d). Interestingly, WT (DM) mice remained in the same temperature zones as WT (non-DM) mice at this age, as distinct from the behavior of 10-week-old mice, while TRPV1^**−/−**^ (non-DM) mice remained at lower temperatures. In addition, the calculated preference temperature was not significantly different among WT (non-DM), WT (DM) and TRPV1^**−/−**^ (non-DM) mice (Fig. 5e).

Because the preference temperature decreased with time in the mice 5 weeks after STZ administration (Fig. 5c), the “travel distance” was also analyzed. We considered the possibility that the travel distance was changed due to locomotor weakening of WT (DM) mice, which might affect their preference temperature. There was no difference in travel distance between WT (non-DM) and WT (DM) mice at 40–60 min (Supplementary Fig. 6a). At all times, TRPV1^**−/−**^ (non-DM) mice were observed to have a longer travel distance than the other mice (Supplementary Fig. 6a). To investigate whether this characteristic of TRPV1^**−/−**^(non-DM) mice is caused by the presence of a temperature gradient, we conducted additional experiments with a Thermal Gradient Ring on WT (non-DM) and TRPV1^**−/−**^ (non-DM) mice in the absence of a temperature gradient (room temperature 23–24 °C). Under these conditions, the travel distance was longer for TRPV1^**−/−**^ (non-DM) mice than for WT (non-DM) mice, while the total travel distance increased for both genotypes (Supplementary Fig. 6b). Finally, we examined the avoidance of noxious low and high temperatures by analyzing “speed” (Supplementary Fig. 6c) because mice characteristically exhibit high speed movements at noxious temperatures. However, speed at low and high temperatures was not significantly different among WT (non-DM), WT (DM), TRPV1^**−/−**^ (non-DM) and TRPA1^**−/−**^ (non-DM) mice, although they showed high speed movement in these noxious temperature zones.

## Discussion

We sought to evaluate changes in temperature sensitivity during the progression of DPN by using rodent behavioral assays. Thermal hyperalgesia in DPN has been reported to be associated with changes in the TRPV1 channels^8,27^, and symptoms such as cold hyperalgesia and numbness are associated with changes in the TRPA1 channels^30,31^. However, few reports have subsequently evaluated the involvement of these channels at the stage of hypoalgesia. By using a newly described behavioral assay, we were able to find behavioral changes that had previously been unrecognized in murine models of DPN. The symptoms of thermosensory impairment of DPN are complicated and many mechanisms have been proposed^2,42–45^. In the present study, we used a STZ-induced model of type 1 DM in mice to evaluate the changes in thermal pain sensitivity in DPN.

A Plantar test and a hot-plate test have been most frequently used to evaluate temperature-dependent behaviors of diabetic mice and rats^5^, and we initially used a Plantar test in the present study. As previously reported, we observed a gradual reduction of thermal sensitivity after administering STZ to mice (Fig. 2a), which appears to mirror the progress of symptoms in patients with DM^9^. Although some studies reported transient thermal hyperalgesia shortly after STZ administration^5–7^, we did not observe such hyperalgesia even with low thermal stimuli. These results suggest that the strain of mice (C57BL6/NCr) used in this study could be suitable for studying the thermal hypoalgesia in peripheral neuropathy. Subsequently, we chose to use the mice 5 weeks after administering STZ (so at 10 weeks old) showing the clear thermal hypoalgesia observed in many studies, confirming the presence of DPN in our murine model. As reported, TRPV1^**−/−**^(non-DM), but not TRPA1^**−/−**^(non-DM) mice showed thermal hypoalgesia^8,9^. Moreover, we performed the Plantar test experiments using TRPV1^**−/−**^ and TRPA1^**−/−**^ mice treated with STZ, and found no difference in PWL in TRPV1^**−/−**^ mice with and without STZ treatment, although the TRPV1^**−/−**^ (non-DM) PWL was significantly longer compared to WT (non-DM) mice even before the STZ treatment. Meanwhile, the PWL of TRPA1^**−/−**^ (non-DM) mice were similar to that for WT (non-DM) mice, but TRPA1^**−/−**^ mice with STZ treatment (TRPA1^**−/−**^ (DM)) exhibited a similar time course to that observed in WT (DM) mice (Fig. 2d). These results suggest that TRPA1 is not involved in STZ-induced DPN. In terms of TRPV1, PWL was not changed by the STZ treatment of TRPV1^**−/−**^ (non-DM) mice, which is consistent with earlier studies^8^ and could have caused misinterpretation of the involvement of TRPV1 in DPN. Indeed, several investigators have reported changes in the expression or function of TRPV1^27,28^. However, we did not observe any significant difference in expression (mRNA or protein level) or function (Ca^2+^-imaging) of TRPV1 (Figs. 3 and 4) 5 weeks after administering STZ although we observed significantly greater expression of TRPV1 at both mRNA and protein levels 2 weeks after administering STZ when we saw no significant difference in thermal responses between WT (non-DM) and TRPV1^**−/−**^ (non-DM) mice in the Plantar test (Fig. 2). The function of TRPA1 was not significantly different between WT (non-DM) and WT (DM) mice (Fig. 4). Moreover, we observed no difference in myelin and nerve structures among WT (non-DM), WT (DM) and TRPV1^**−/−**^ (non-DM) mice (Supplementary Fig. 4) using TEM. Therefore, we concluded that the changes observed in the Plantar test 5 weeks after STZ administration is not caused by the obvious structure damages detected at a TEM level. These results suggest that TRPV1 and TRPA1 is not involved in the thermal hypoalgesia observed in WT (DM) mice and prompted us to seek for other types of assay to evaluate the temperature-dependent phenotypes.

We chose to use a newly described Thermal Gradient Ring test of mouse behavior. The ring-shaped temperature gradient system allowed us to determine accurate values for spent time or speed because mice can freely access all the temperature zones. Many studies of temperature-dependent behaviors have used a Plantar test or a hot-plate test, both of which can examine the extent of thermal nociception^37–39^. However, it is not possible to determine temperature preference with those tests. A linear thermal-gradient system solves this problem. However, mice have a habit of hiding in corners, which could prevent us from analyzing mice behavior in either a cold or hot zone with corners. The Thermal Gradient Ring used in the present study excludes the contribution of open field anxiety, and so we can determine mouse reactions to thermal differences by measuring various other parameters, such as travel distance and speed. With this Thermal Gradient Ring, we found two different points between WT (non-DM) and WT (DM) mice (5 weeks after administering STZ); a shift in temperature-dependent curves of spent time and preference temperature calculated as the mean values from the spent time and the zone temperatures (Fig. 5b,c). Temperature preference was shifted to lower temperatures in mice with WT (DM) when compared to WT (non-DM) and TRPA1^**−/−**^ (non-DM) mice. Interestingly, the temperature-dependent curves of spent time were similarly shifted to low temperatures in age-matched TRPV1^**−/−**^ (non-DM) mice, while temperature preference in TRPV1^**−/−**^ (non-DM) mice was similar to that in WT (non-DM) mice. Although the preference temperature was calculated as the mean value from the spent time in the various temperature zones, an apparent difference in the two parameters indicates that calculated preference temperature reflects the mouse behavior in all the temperature zones. That WT (DM) mice avoided noxious high temperatures as did WT (non-DM) mice (expanded inset in Fig. 5b) could explain, at least in part, the difference in preference temperature between WT (DM) and TRPV1^**−/−**^ (non-DM) mice. This is consistent with data showing that expression and function of TRPV1 are not significantly different between WT (DM) and WT (non-DM) mice. Although it is clear that WT (DM) mice preferred to remain at the lower temperature zones, we currently cannot explain this phenotype. We note that TRPA1^**−/−**^ (non-DM) mice exhibited temperature-dependent curves of spent time and preference temperature that are similar to those of WT (non-DM) mice, suggesting that TRPA1 is not involved in the temperature-dependent behavior, at least in the temperature zones that we examined.

It has been generally reported that thermal pain sensation in DPN is related to changes in expression or function of TRPV1 or TRPA1. However, in the current study, WT (DM) mice showing reduced thermal nociceptive behaviors in the Plantar test avoided nociceptive high temperatures as did WT (non-DM) mice in the Thermal Gradient Ring. WT (DM) mice preferred lower temperatures than WT (non-DM) mice 5 weeks after administering STZ. Therefore, this study suggests that TRPV1 and TRPA1 could not be associated with thermosensory impairment in DPN. Based on these results, it is possible that the thermal sensing function in DPN is complicated, and several factors and differences in the stage of neuropathy progression could be involved, which is consistent with the progress of DPN being affected by multiple factors^2,42–44^. In the Plantar test, it is possible that multiple applications of infrared laser stimuli may cause anticipatory behavior at temperatures lower than the threshold for TRPV1 activation.

In addition, we can consider the contribution of preference with the Thermal Gradient Ring because human and mouse behaviors can generally be determined by a balance between avoidance and preference. These two factors interact mutually and are difficult to discriminate. Therefore, temperature-related symptoms in DPN are best investigated by focusing not only on avoidance behaviors, but also behavioral changes based on preference. Analysis with Thermal Gradient Ring may be suitable for detecting subtle changes in temperature-dependent DPN-related behavior occurring at the earlier stage of DPN. Accordingly, we propose approaches with multiple behavioral methods to analyze the progression of DPN in response to thermal stimuli.

## Methods

### Mice

We used 5–10-week-old C57BL/6NCr male mice (SLC, Shizuoka, Japan) as the wild type (WT), and TRPV1-deficient (TRPV1^**−/−**^) and TRPA1-deficient (TRPA1^**−/−**^) male mice maintained on a C57BL/6NCr background^46^. Mice were housed in standard cages and maintained under a 12-h light/dark cycle at an ambient temperature of 24 ± 2 °C with access to food and water ad libitum. All the animal care and experimental procedures were approved by our Institutional Animal Care and Use Committee and followed the National Institutes of Health and National Institute for Physiological Sciences guidelines (21A008), and carried out in compliance with the ARRIVE guidelines.

### Induction of diabetes with STZ and measurement of serum insulin levels in the murine model

Diabetes was induced in mice by administering a single intraperitoneal dose of 150 mg/kg STZ (Sigma-Aldrich) prepared freshly in 0.02 M citrate buffer (pH 4.5) after a 24-h fasting period when they became 5 weeks old. WT (non-DM), TRPV1^**−/−**^ (non-DM) and TRPA1^**−/−**^ (non-DM) mice received an equal volume of citrate-buffer vehicle. One week after administering STZ, the mice with consequent blood glucose concentrations of > 400 mg/dL were selected as WT (DM), TRPV1^**−/−**^ (DM) and TRPA1^**−/−**^ (DM) mice. Blood glucose levels were measured by Glutest Neo (Sanwa Kagaku Kenkyusho, Nagoya, Japan). Serum insulin concentration was measured by collecting blood from mice by cardiac puncture into a heparin-containing tube, collecting the supernatant immediately after centrifugation, freezing it at −80 °C and transporting it at low temperature to a testing contractor (Nikken Seil, Tokyo, Japan). The limit of detection for insulin levels was < 0.1 ng/mL.

### Plantar test

While the mice were 5–10 weeks old, hind paw withdrawal response to thermal stimuli of radiant heat was measured using a Plantar test (Catalog No. 57820; Ugo Basile, Comerio, Italy)^47,48^. The PWL at 5 weeks of age was measured a few days before STZ and the buffer administration. We adjusted the two kinds of IR intensities to a PWL baseline of about 7 s (IR = 40) and 12 s (IR = 20). After 30 min acclimation, paw withdrawal latencies (PWL) were measured 6–8 times per session, separated by a minimum interval of 5 min. Paw withdrawals due to locomotion or weight shifting were not counted. Data are expressed as paw withdrawal latency in seconds.

### qRT-PCR

Mice were killed after anesthesia with isoflurane, and dorsal root ganglia (DRG) were quickly harvested and placed on ice. The tissue was then immediately immersed in RNAlater Stabilization Solution (Invitrogen). After temporarily storing at 4 °C, RNAlater was removed, and an appropriate volume of Isogen II (Nippon Gene, Tokyo, Japan) was added to homogenize the ganglia with a Biomasher II apparatus (Nippi, Tokyo, Japan); they were completely homogenized and cells were lysed on ice. Then, total RNA was collected using Ethachinmate (Nippon Gene, Tokyo, Japan) and 75% isopropanol and RNA concentration was assayed using a NanoDrop One Microvolume UV–Vis Spectrophotometer (Thermo Fisher Scientific, United States). The RNA was reverse transcribed into cDNAs with ReverTra Ace qPCR Master Mix (Toyobo, Osaka, Japan) according to the manufacturer’s protocol. TRPV1, TRPA1, and 36B4 mRNA levels were assayed using a StepOnePlus Real-Time PCR System (Applied Biosystems) with SYBR Green Real Time PCR Master Mix Plus (Toyobo, Osaka, Japan) according to the manufacturer’s protocol. All data were analyzed using StepOne software (version 2.3; Life technologies).

The primer sequences used for qRT-PCR were as follows: TRPV1 (NM_001001445), 5′-CCCGGAAGACAGATAGCCTGA -3′ (forward) and 5′-TTCAATGGCAATGTGTAATGCTG-3′ (reverse); TRPA1 (NM_177781), 5′-GTCCAGGGCGTTGTCTATCGG -3′ (forward) and 5′-CGTGATGCAGAGGACAGAGAT-3′ (reverse); 36B4 (NM_007475.5), 5′-AGATTCGGGATATGCTGTTGGC-3′ (forward) and 5′-TCGGGTCCTAGACCAGTGTTC-3′ (reverse).

### Western blotting

DRG were isolated and rinsed immediately in ice-cold phosphate-buffered saline (PBS; calcium- and magnesium-free) and put into an appropriate amount of protein lysis buffer (25 mM Tris–HCl [pH 7.6], 150 mM NaCl, 0.1% sodium dodecyl sulfate [SDS], 1% Nonidet P-40, and 1% protease inhibitor), and homogenized using a Biomasher II apparatus. The homogenates were then placed on ice for 30 min to ensure complete lysis. Subsequently, the homogenates were centrifuged at 15,000 ×*g* for 30 min at 4 °C and the supernatant was transferred to a new centrifuge tube. After measuring the protein concentration of each sample using a BCA Assay Kit (catalog No. 297-73101, Fujifilm Wako Chemicals), equal amounts of protein from the DRG were denatured at 95 °C for 5 min and electrophoresed on 4%–12% SDS–polyacrylamide gel. The proteins were transferred onto a poly(vinylidene fluoride) membrane. Nonspecific binding sites on the membranes were blocked using Tris-buffered saline (TBS) supplemented with 0.05% Tween 20 (Takara Bio, Shiga, Japan) (TBS-T) and bovine serum albumin (BSA) for 1 h at room temperature (RT), and incubated overnight at 4 °C with rabbit anti-TRPV1 antibody (1∶3000, Dr. Kido, Saga Medical School Faculty of Medicine, Saga University) and rabbit anti-β-actin antibody (1∶3000, Cell Signaling Technology). Then anti-rabbit IgG HRP-linked secondary antibodies (1∶1000, Cell Signaling Technology) were incubated with the membranes for 1 h at RT. Between respective steps, the immunoblots were rinsed with TBS-T 3 times for 10 min each time. All protein bands were labeled using an ECL kit (Amersham, United Kingdom) and then visualized using an ImageQuant LAS 4000 system (General Electric). The densities were normalized with respect to the β-actin level.

### Isolation of mouse dorsal root ganglion neurons

According to a published method^49^ with some minor modifications described as follows, DRG were isolated from 5 to 10 week old mice. In brief, resected DRG were collected in PBS (calcium- and magnesium-free) on ice, and then the tissues were incubated with 725 μg of collagenase type IX (catalog No. C9407, Sigma-Aldrich) in 250 μL of Earle’s balanced salt solution (Sigma-Aldrich) containing 10% fetal bovine serum, MEM vitamin solution (1:100, Sigma-Aldrich), penicillin–streptomycin (1:200, Life Technologies), and GlutaMax (1:100, Life Technologies) at 37 °C for 30 min. Next, the DRG neurons were dissociated by triturating the suspension through a fire-polished Pasteur pipette and filtering it through a 70 μm cell strainer (Flowmi). The isolated neurons were placed on 12 mm diameter coverslips (Matsunami, Osaka, Japan) with 20 μL of Earle’s balanced salt solution and used for experiments within 2 h of isolation, maintaining them at 37 °C in a chamber under a humidified atmosphere of 95% O_2_ and 5% CO_2_.

### Calcium imaging

Ca^2+^ transients were measured in isolated cultured DRG neurons incubated with 5 mM Fluo-2-AM (Molecular Probes, Invitrogen) for 20 min at 37 °C, and DRG were mounted in an open chamber and superfused with bath solution. The extracellular standard bath solution contained 140 mM NaCl, 5 mM KCl, 2 mM MgCl_2_, 2 mM CaCl_2_, 10 mM HEPES, and 10 mM glucose at pH 7.4, adjusted with NaOH. Cytosolic free Ca^2+^ concentrations were measured by dual-wavelength Fura-2 microfluorometry with excitation at 340/380 nm and emission at 510 nm. Fura-2 fluorescence was recorded with a CCD camera, CoolSnap ES (Roper Scientific/Photometrics). Data were acquired using imaging processing software IPlab (Solution Systems, Funabashi, Japan) and analyzed with ImageJ 1.53 (NIH). At the end of each experiment, ionomycin (5 μM) was applied in the presence of 20 mM extracellular CaCl_2_ to obtain saturating levels of Ca^2+^ influx as F_max_. The population that did not respond to either molecular stimulus, responded to AITC alone, capsaicin alone, and the population responding to both stimuli were determined by the number of neurons responding to capsaicin and/or AITC divided by the number of neurons responding to ionomycin and expressed as a percentage.

### Light and electron microscopy

Distal sciatic nerve tissue was prepared for imaging as previously described with slight modifications^50^. WT (non-DM), WT (DM) and TRPV1^**−/−**^ (non-DM) mice (5 weeks after STZ administration or the same age) were perfused with 2.5% glutaraldehyde and 4% paraformaldehyde in 0.1 M phosphate buffer. We collected sciatic nerves from the same position for all mice, and these tissues were post-fixed for 4 h, and maintained at 4 °C overnight. The samples were post-fixed in cold 2% OsO4 in PBS for 60 min, dehydrated in a graded ethanol series and acetone and embedded in Quetol 812 epoxy resin (Nisshin EM Co.). The resin was incubated at 70 °C for 3 nights to ensure polymerization. Prior to TEM observation, semithin, 1 μm-thick sections were cut and stained with 1% toluidine blue for examination by light microscopy (AX80; Olympus). Ultrathin sections (70 nm-thick) were prepared with an ultramicrotome (ULTRACUT S, Reichert-Nissei) and stained with uranyl acetate and lead citrate. The ultrathin sections were observed by TEM (HT7700; Hitachi High-Tech). Image analysis was performed with Image J software. The g-ratios were calculated by dividing the axon diameter by the diameter of the axon including the myelin. The diameter was calculated by the measured perimeter divided by π.

### Behavioral assay with the thermal gradient ring

The Thermal Gradient Ring (Catalog No. 35550; Ugo Basile) is an apparatus with 45 cm inner diameter, 57 cm outer diameter, and 24 cm height. A camera is located on the upper side of the apparatus, which includes an infrared camera and an infrared transmissive inner wall. The cooling and heating devices were set so that the surface temperature range of the apparatus was from 10 to 55 °C. Floor surface temperature was monitored using a thermometer (HFT-51, Anritsu, Japan). Behavioral assays were performed between 9:00 and 17:00. All mice were acclimated for 30 min in the thermal gradient apparatus with its floor at room temperature (23–24 °C) before the day of the thermal gradient test. Mice were placed individually in the device in an innocuous mid-temperature zone. The behavioral data was videotaped for 60 min and analyzed as “spent time”, “travel distance” or “speed” automatically using ANY-maze software. We defined “preference temperature” as the mean value using the zone temperature and “spent time”.

### Statistic and reproducibility

Data are presented as means ± SEM. Statistical analysis was conducted using GraphPad Prism 9.2.0 (GraphPad Software, United State). Significant changes were identified using a two-tailed *t* test, at 95% confidence interval, or one-way ANOVA and two-way repeated measures ANOVA followed by a Bonferroni post hoc test with *p* < 0.05 considered as significant (*p*: * < 0.05, ** < 0.01, *** < 0.01).

## Supplementary Information

Supplementary Information.

Supplementary Information.

Supplementary Information.

Supplementary Information.

Supplementary Information.

Supplementary Information.

Supplementary Information.

## Data Availability

All data and materials used in the analysis are available in the main text or Figures and Supplemental Figures. Other data or information that supports the findings of this study are available from the corresponding author upon reasonable request.
