# Supplementary figures and images for: Thermal gradient ring reveals thermosensory changes in diabetic peripheral neuropathy in mice
Source: Sci Rep. 2022 Jun 13;12:9724. doi: 10.1038/s41598-022-14186-x (PMC9192750; doi:10.1038/s41598-022-14186-x)

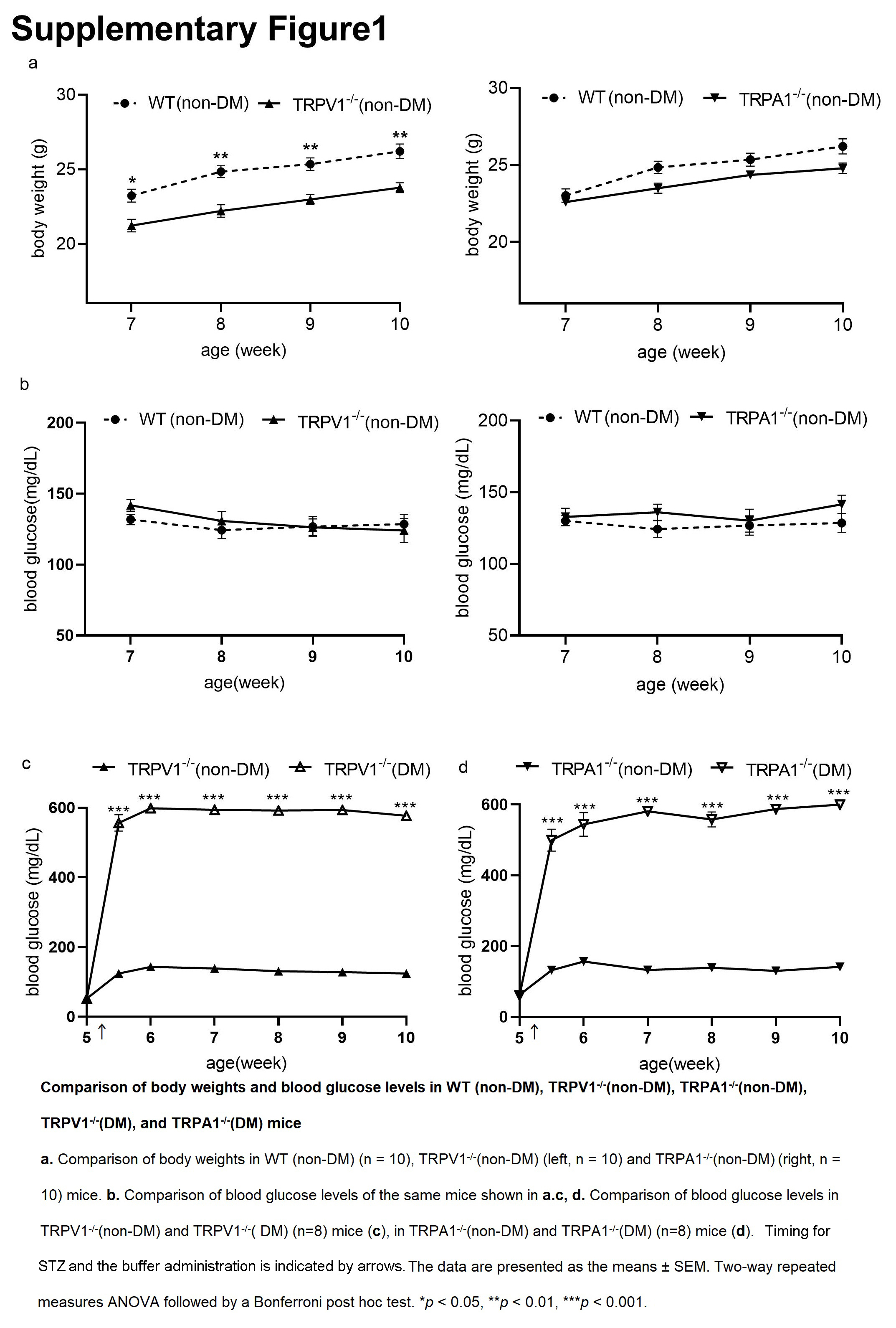

Supplement: Supplementary file 1 — Supplementary Information. [file 41598_2022_14186_MOESM1_ESM.jpg]

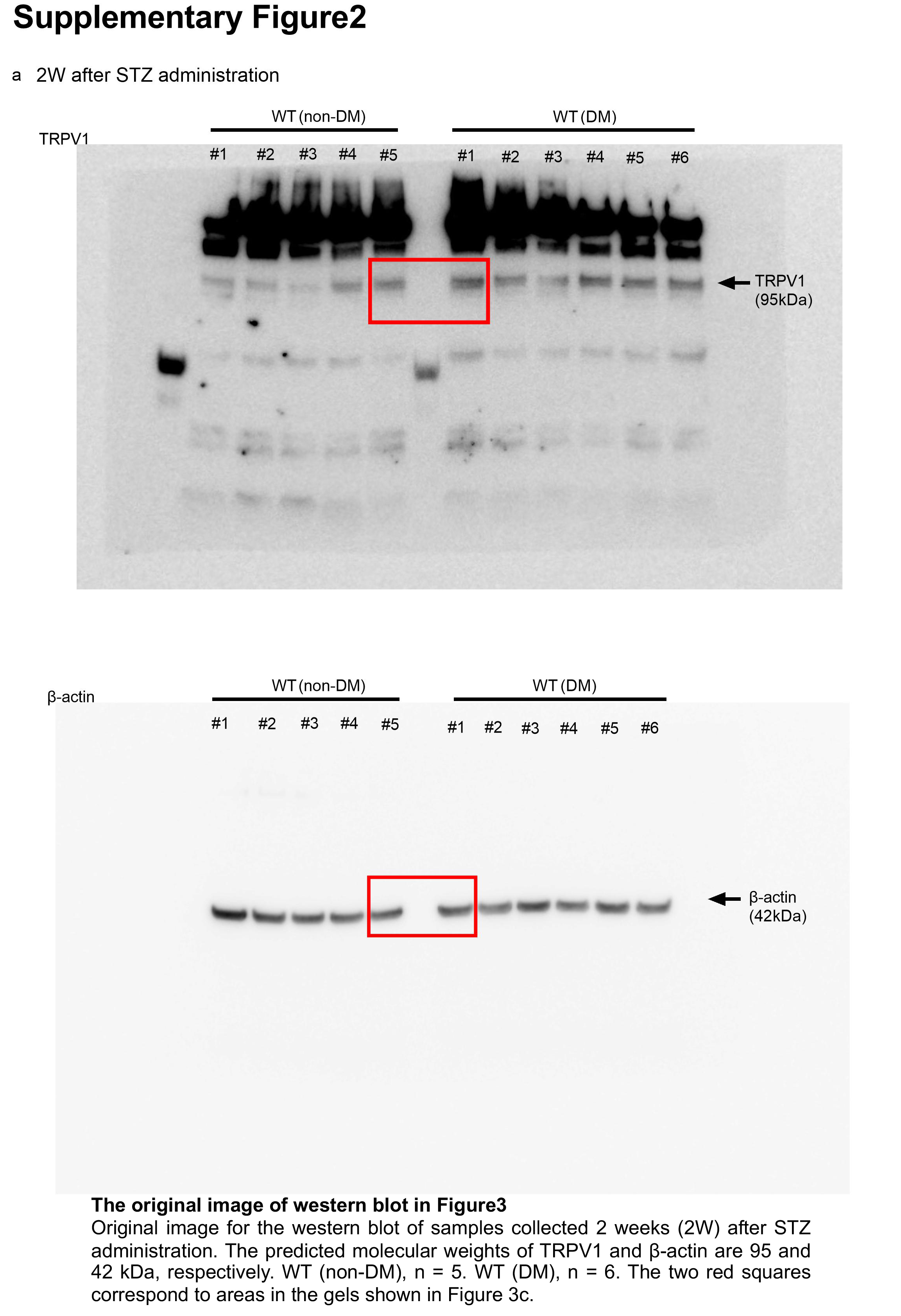

Supplement: Supplementary file 2 — Supplementary Information. [file 41598_2022_14186_MOESM2_ESM.jpg]

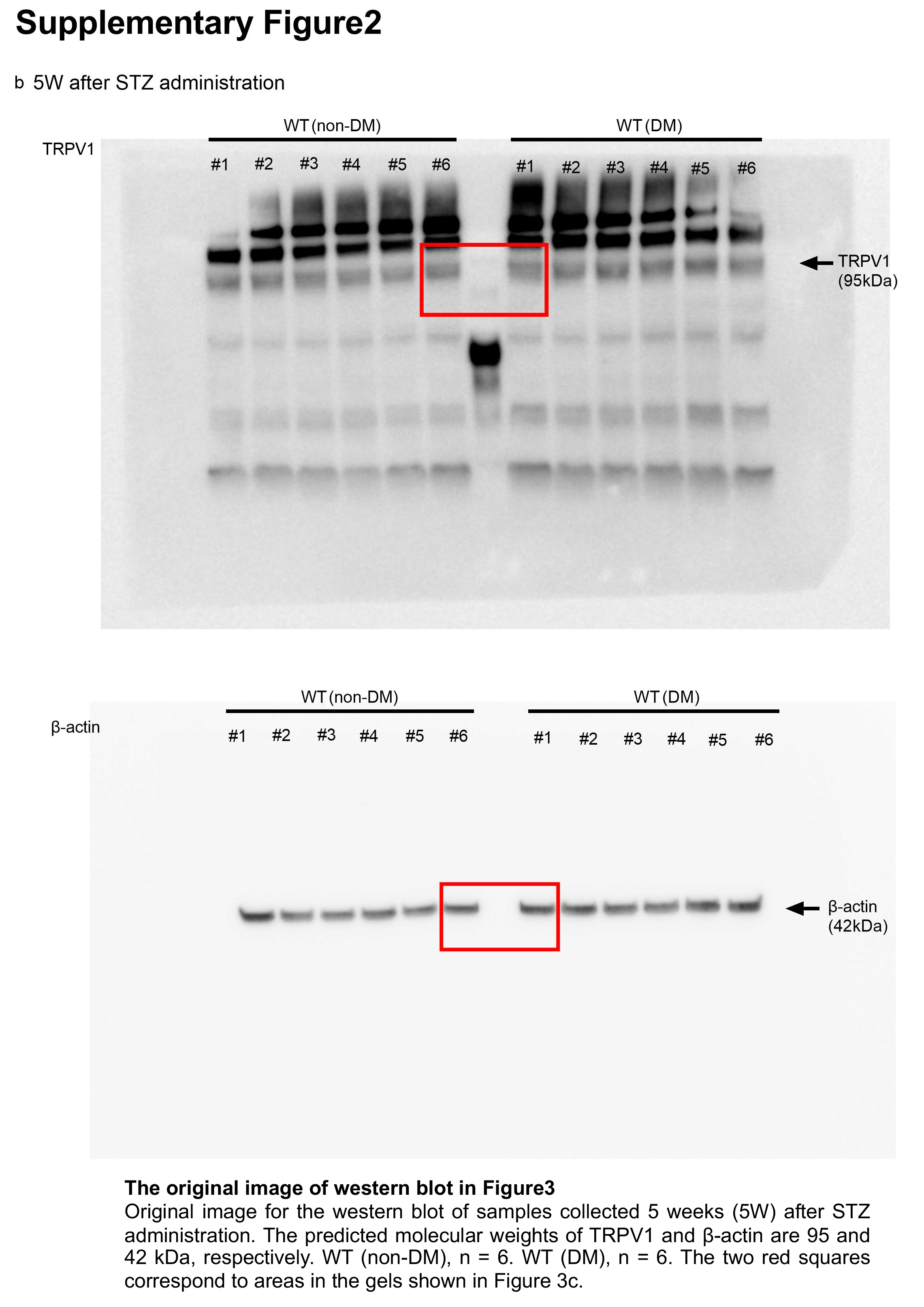

Supplement: Supplementary file 3 — Supplementary Information. [file 41598_2022_14186_MOESM3_ESM.jpg]

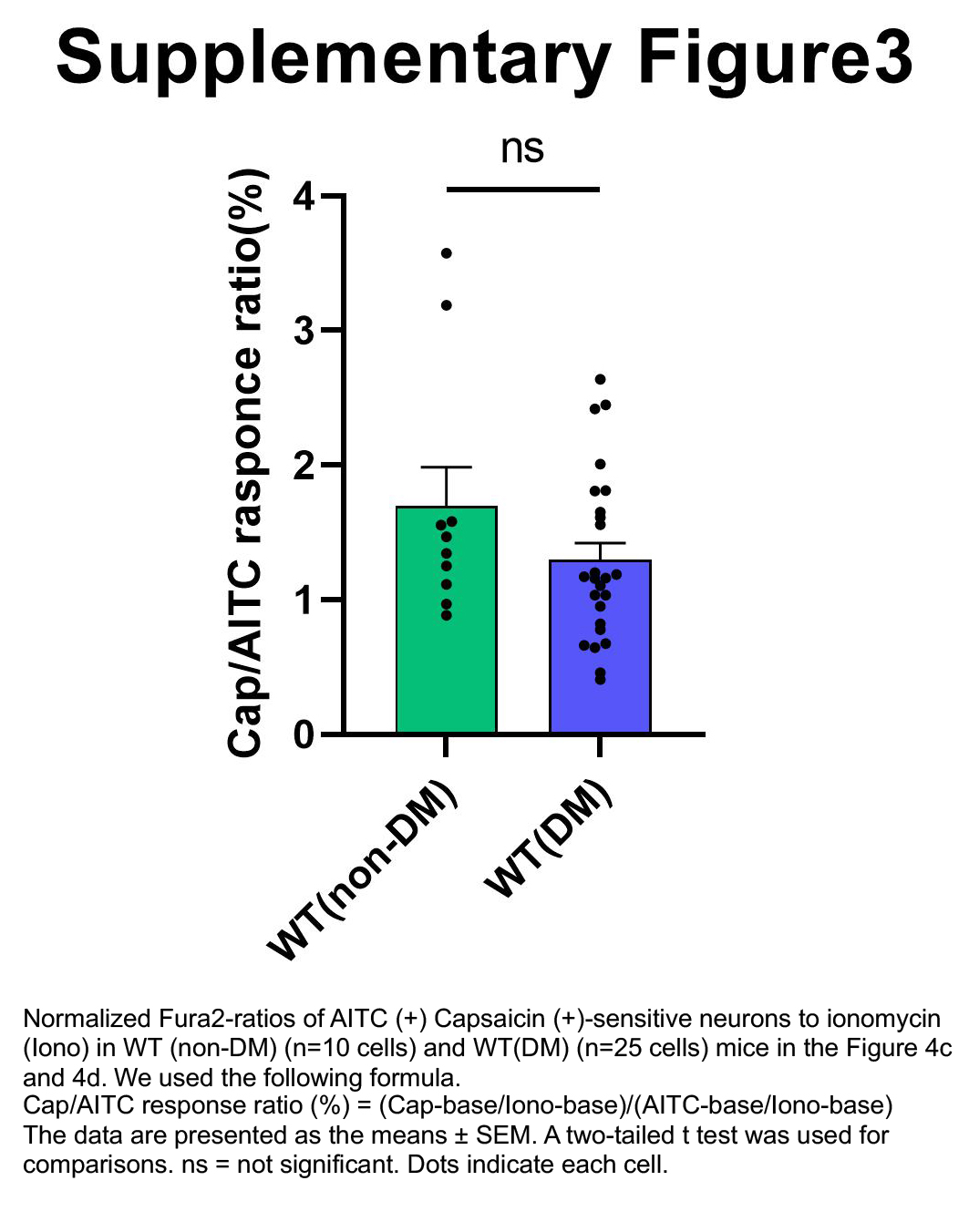

Supplement: Supplementary file 4 — Supplementary Information. [file 41598_2022_14186_MOESM4_ESM.jpg]

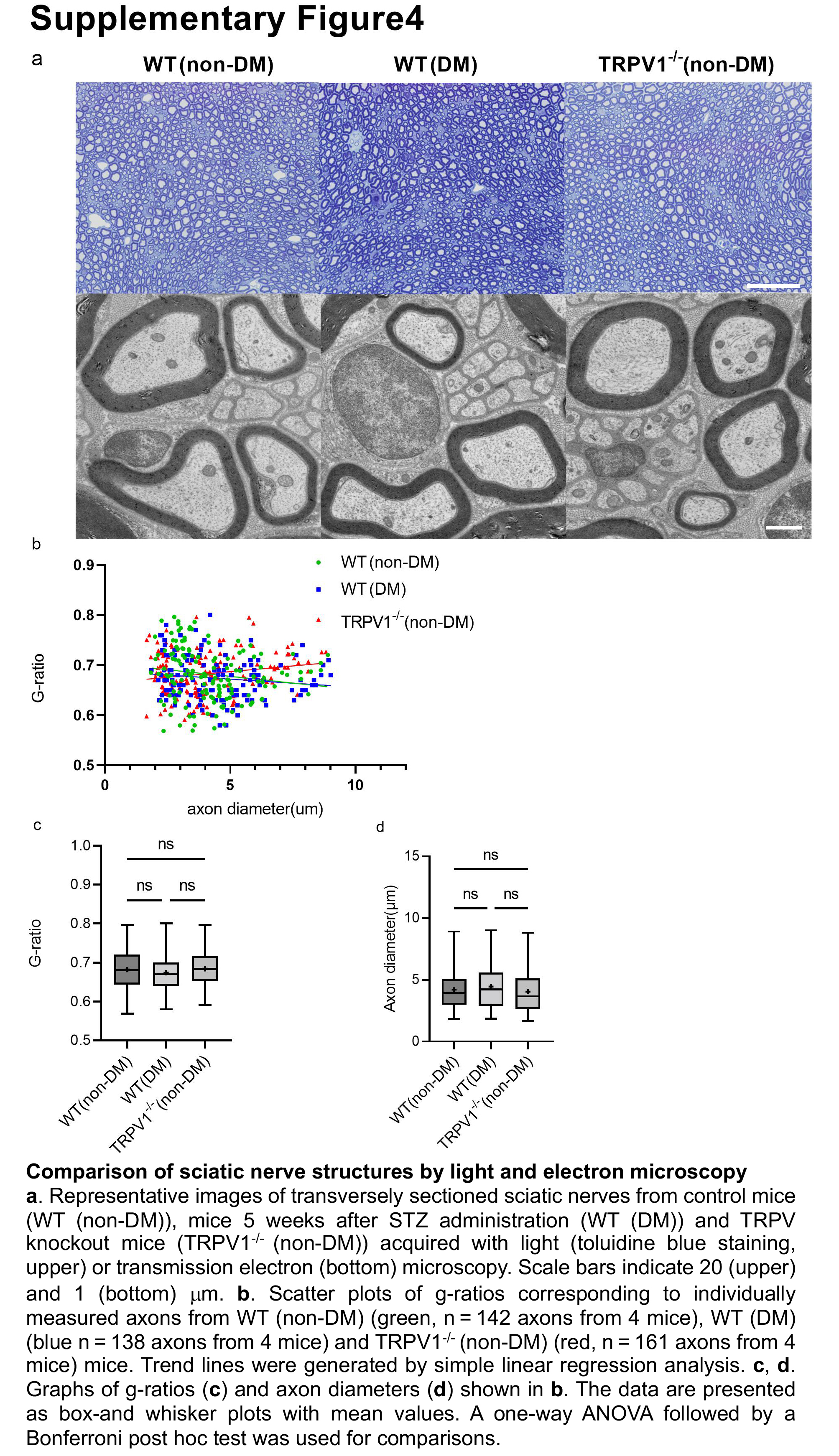

Supplement: Supplementary file 5 — Supplementary Information. [file 41598_2022_14186_MOESM5_ESM.jpg]

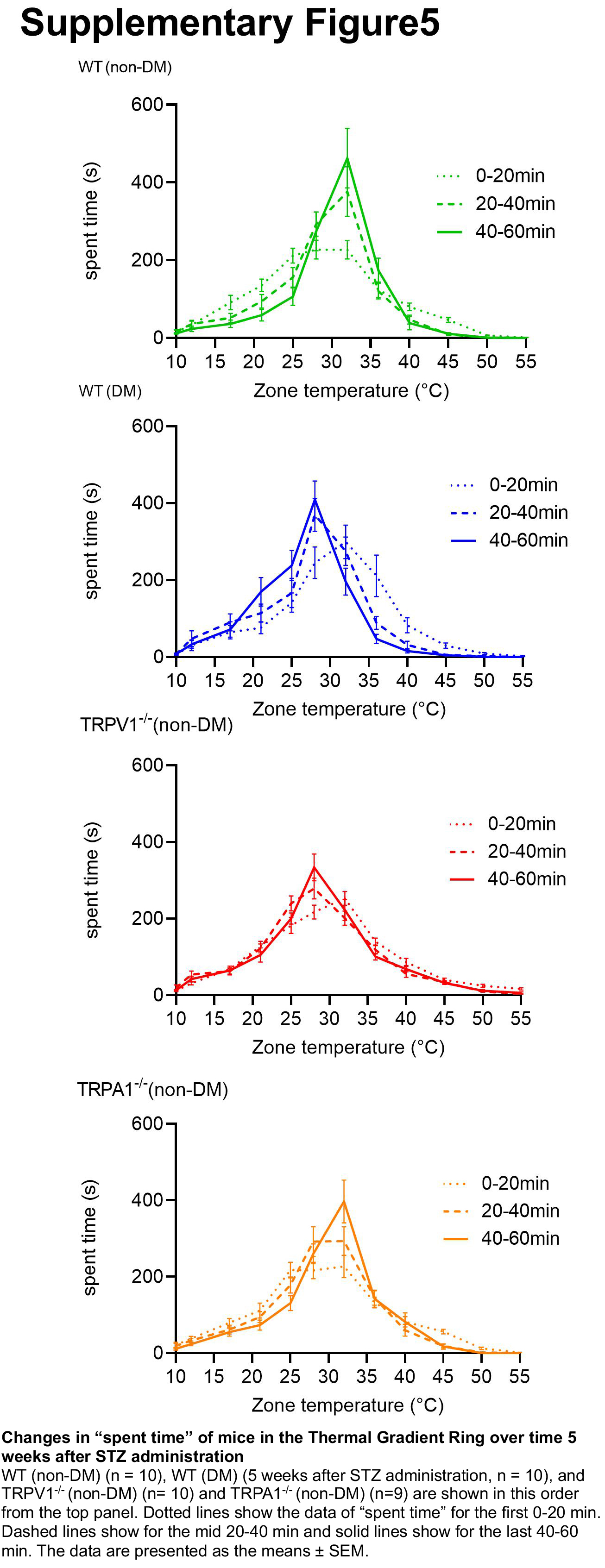

Supplement: Supplementary file 6 — Supplementary Information. [file 41598_2022_14186_MOESM6_ESM.jpg]

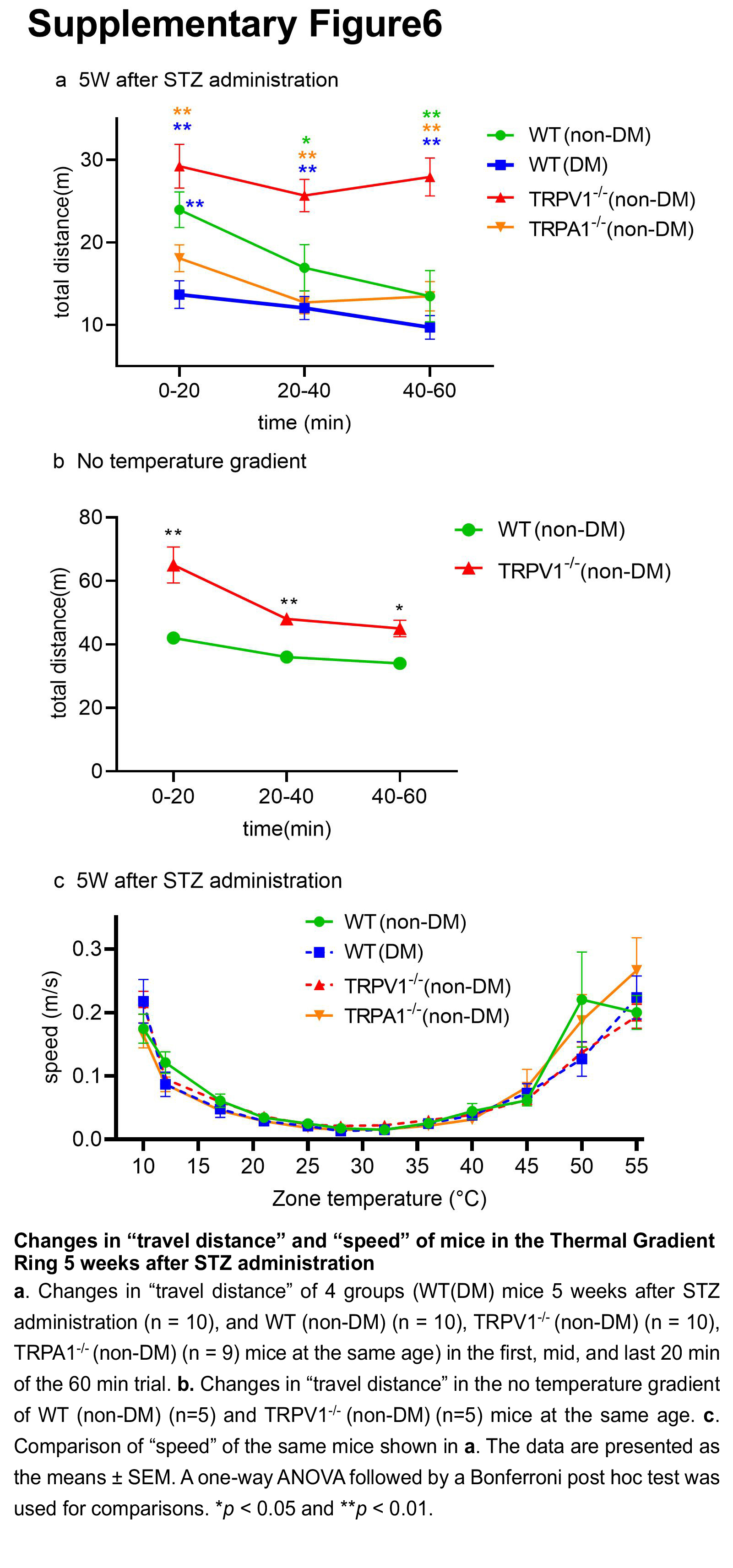

Supplement: Supplementary file 7 — Supplementary Information. [file 41598_2022_14186_MOESM7_ESM.jpg]
